# Brodie's Abscess in a Patient Presenting with Sickle Cell Vasoocclusive Crisis

**DOI:** 10.1155/2015/429876

**Published:** 2015-07-28

**Authors:** Onyekachi Henry Ogbonna, Yonette Paul, Hasan Nabhani, Adriana Medina

**Affiliations:** Division of Hematology and Oncology, Howard University Hospital, Washington, DC 20060, USA

## Abstract

First described by Sir Nicholas Brodie in 1832, Brodie's abscess is a localized subacute or chronic infection of the bone, typically seen in the metaphases of long bones in children and adolescents. The diagnosis can prove to be enigmatic due to absence of clinical signs and symptoms of systemic disease. We report a very interesting case of Brodie's abscess masquerading as sickle cell vasoocclusive crisis in a 20-year-old female with sickle cell disease and review the literature.

## 1. Introduction

Brodie's abscess is a rare form of osteomyelitis. It involves a subacute or chronic infection of the bone with development of a localized abscess, usually within the metaphysis of long bones. The tibia is the most common bone involved and staphylococcus aureus is the most common organism identified. The diagnosis is usually made with the help of radiological imaging since signs or symptoms of systemic disease are frequently absent. We report a case of Brodie's abscess in a patient presenting with bony pain that was initially attributed to sickle cell vasoocclusive crisis.

## 2. Case Report

A 20-year-old female patient with hemoglobin-SS sickle cell disease, osteonecrosis of the right hip with chronic right hip pain, and asthma presented complaining of pain in both thighs for 5 days. The pain began after ice-skating but she denied any direct trauma to her limbs. On presentation, she was febrile and tachycardic, with leukocyte count of 25.3 × 10^9^/L and hemoglobin of 7.0 g/dL. There was no tenderness to palpation of the hips bilaterally and no tenderness to palpation of the joints, but an ill-defined swelling to the right anterior thigh was noted which the patient reported was chronic.

An initial diagnosis of sickle cell vasoocclusive painful crisis with systemic inflammatory response syndrome was made, and sepsis work-up was done. She was started on analgesics and intravenous fluids and also received a simple blood transfusion. On hospital day (HD) 2, she was noted to have persistent fever and leukocytosis and so was started on intravenous Levofloxacin empirically. At that time, her left thigh pain appeared to be worsening, with a new area of swelling and warmth but no tenderness. X-rays of the left hip and left thigh were done, which showed heterogeneous appearance of the proximal left femoral shaft, which was thought to be likely secondary to sickle cell osteodystrophy, infarct, or infection.

**Figure 1:**
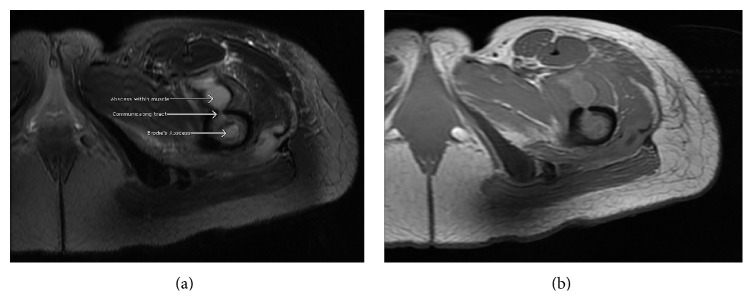
Axial views of MRI showing the Brodie's abscess in the left proximal femur, with a break in the cortex and a tract communicating with the abscess and overlying muscle. (a) T2-weighted image; (b) T1-weighted image.

The patient's blood cultures obtained on admission had shown no growth and her urine culture was negative. Her chest X-ray showed no evidence of pneumonia. However, clinically, she did not appear to be improving, with persistent pyrexia and leukocytosis. Her C-reactive protein levels were elevated at 7.8 mg/dL, for which her dose of Levofloxacin was increased and Vancomycin was added. Due to increasing suspicion for osteomyelitis, an MRI of the left lower extremity was done. It demonstrated 2 areas of loculated fluid collection: the first was in the proximal left thigh anteriorly, in the quadriceps muscle, measuring approximately 1.8 × 2.0 × 4.0 cm. The second was in the proximal left thigh laterally, between the quadriceps muscle and subcutaneous tissue, and measured approximately 2.8 × 3.0 × 10.0 cm. These areas had low signal intensity on T1 weighted imaging and high signal intensity on fluid sensitive sequences. An abscess was noted within the left proximal femur, with a communicating tract extending from the abscess within the bone to the overlying muscle and subcutaneous tissue (Figure 1). The radiological findings were consistent with a Brodie's abscess.

Orthopedics consultation was sought and the patient subsequently underwent incision and drainage of the left thigh abscesses in the operating room. A large amount of pus was found within the thigh muscles, which extended down into the bone. Two defects were also identified in the bone where the pus appeared to be extruding from. The area was debrided and irrigated, a Jackson Pratt drain was left in situ, and the wound was closed. Clindamycin was added to her antibiotic regimen. Postoperatively, leukocytosis began trending down slowly and the patient became afebrile. She was started on physical therapy for early mobilization, which she tolerated well. Wound cultures obtained during the incision and drainage surgery showed no growth of organisms. On post-op day (POD) 3, the drain was removed.

Her postoperative course was subsequently complicated by persistent fevers that started on POD 4. High sensitivity C-reactive protein (hs-CRP) was obtained and noted to be significantly elevated at 101.54 mg/L (normal < 0.5 mg/L). CT scan of bilateral lower extremities showed anterior thigh abscesses, involving multiple muscle groups of both lower extremities. Her antibiotics were switched to Imipenem. She was taken back to the operating room where she underwent incision and drainage of the left and right thigh abscesses. A copious amount of purulent material was found within the anterior soft tissue of the right thigh, which communicated with a hole in the proximal femur. Exploration of the left thigh revealed small amounts of pus and a hematoma around the femur, as well as the previously identified defects in the bone. Jackson Pratt drains were left in the right and left thighs. Wound cultures obtained were negative again, with no organisms seen on gram stain.

Unfortunately, she continued to spike fevers and leukocytosis persisted. Repeat blood cultures, urine culture, and chest X-ray were negative. A repeat CT scan of the lower extremities was done, showing residual abscess in the iliopsoas muscle and vastus lateralis muscle of the left thigh and in the right thigh. Antibiotics were switched to Vancomycin and Levofloxacin but then subsequently changed to Linezolid and Piperacillin-Tazobactam. Percutaneous ultrasound-guided drainage of the residual bilateral thigh abscesses was undertaken: 3 right thigh abscesses were identified and approximately 4.5 mL of purulent fluid was drained; approximately 4.0 mL of fluid was aspirated from the left thigh fluid collection. Postprocedure CT scan showed minimal residual abscess fluid. Jackson Pratt drains were kept in situ and continued to drain moderate amounts of serosanguineous fluid.

Three days after percutaneous drainage, the patient remained afebrile and her leukocyte count was consistently trending downwards. She had a repeat CT scan of the lower extremities, which showed decreased fluid collection and soft tissue swelling of the thighs compared with prior studies, bony changes consistent with sickle cell osteodystrophy, and chronic osteomyelitis. She was discharged to a long-term acute care hospital to facilitate continued intravenous antibiotic therapy with Vancomycin and Piperacillin-Tazobactam for at least 6 weeks. She presented for follow-up in infectious disease clinic 3 weeks later and was noted to be doing well with no fever episodes and significantly decreased pain. Repeat hs-CRP was significantly lower at 33.75 mg/L. She was continued on antibiotics for the proposed course.

## 3. Discussion

Brodie's abscess can be defined as a confined abscess within bone surrounded by a sclerotic rim of dense bone [1]. It is a rare form of subacute osteomyelitis that was first described by Sir Benjamin Brodie in 1832. He had identified 8 cases of osteomyelitis in the tibia in which the infection was well circumscribed, with subacute or chronic presentation (rather than acute), and without evidence of systemic illness or fever [2]. The abscess is usually localized within the metaphysis of long bones although diaphyseal involvement has been reported in some cases. Diaphyseal involvement is thought to be more common in adults [3]. The tibia is the most commonly involved bone but cases involving the femur, fibula, ulna, radius, and talus have also been reported. Brodie's abscesses are mostly seen in children and adolescents with an average age of 19.5 years and there is a slight male predominance (male to female ratio of 3 : 2) [1]. The most commonly identified pathogen is staphylococcus aureus, although in about 25% of cases, no organism is identified [1]. Other pathogens like* Pseudomonas aeruginosa*,* Klebsiella* spp., and* Salmonella typhi* have been reported in the literature [3, 4].

Patients may present complaining of deep pain in the affected area lasting for weeks to months [2] in the absence of trauma and are typically afebrile. Laboratory findings usually include a normal white blood count and differential. Erythrocyte sedimentation rate and C-reactive protein levels may be normal or could be elevated in about 50% of cases suggesting an active infection [1]. The diagnosis is made primarily by radiological imaging: plain X-rays reveal an area of central lucency surrounded by a sclerotic margin [2]. Computed tomography (CT) shows features seen in plain X-ray but enhances the visualization of sinus tracts and eccentrically sited sequestra that help in detecting infection [5]. Magnetic resonance imaging (MRI) shows characteristic features including the pronounced rim enhancement, the “double line” sign, and the “penumbra” sign [5] and reveals the well-circumscribed nature of the lesion as compared to more extensive involvement seen in diffuse osteomyelitis [1]. SPECT-CT is a hybrid imaging technique that has also been used in the diagnosis of Brodie's abscess [6]. It integrates single-photon emission computed tomography (SPECT) and routine CT, allowing for the direct fusion of morphological and functional information. The correlation of functional imaging to anatomical data increases the sensitivity and specificity of detecting and localizing an infectious process or tumor by avoiding the false positives and equivocal results seen with scintigraphy [6, 7]. Brodie's abscess has been radiologically described as a “geographic, lytic lesion with moderately well- or well-defined edges and without soft tissue mass, matrix, cortical destruction, or bony enlargement occurring in the long bones” [1]. Histopathologic analysis of surgical specimen can also aid in the diagnosis [5].

Treatment of Brodie's abscess typically involves surgical debridement and prolonged antibiotic therapy, usually lasting about six weeks. The surgical debridement should be thorough, with removal of all necrotic, nonviable bones and any infected granulation tissue to prevent reinfection [2]. Debridement also provides specimen to help identify causal pathogen. Antibiotic selection should be guided by culture data if available. Bone grafting should be considered for patients with lesions greater than 3 cm or those thought to be at risk for fracture [1].

Our case is unique due to the confounding sickle cell vasoocclusive crisis our patient presented with which could explain her pain. Some patients with sickle cell disease also report chronic baseline manageable pain in the limbs even when not in an acute crisis. Her thigh pain was initially attributed to vasoocclusive crisis and the diagnosis of Brodie's abscess was made only after magnetic resonance imaging of her lower extremities was obtained for persistent fevers. To our knowledge, only one prior case of Brodie's abscess masquerading as sickle cell vasoocclusive crisis has been reported. The patient was diagnosed via SPECT-CT imaging and treated with surgical debridement and IV antibiotics [6]. Brodie's abscess should therefore be considered in the differential for a patient with sickle cell disease presenting with vasoocclusive pain crisis.

## 4. Conclusion

Brodie's abscess is a rare subacute osteomyelitis that can be quiescent in presentation, without fever or evidence of systemic infection. Clinical suspicion is therefore necessary to make the diagnosis. The diagnosis is usually made with radiological imaging and the condition is treated with surgical debridement and antibiotics. Brodie's abscess should be considered in differential diagnosis of a sickle cell patient complaining of subacute bony pain.

## References

[1] Johnson J. W., Bindra R. R. (2008). Brodie's abscess of the distal radius: an unusual complication after percutaneous pinning. *Hand*.

[2] Alter S. A., Sprinkle R. W. (1995). Brodie's abscess: a case report. *The Journal of Foot and Ankle Surgery*.

[3] Olasinde A. A., Oluwadiya K. S., Adegbehingbe O. O. (2011). Treatment of brodie's abscess: excellent results from curettage, bone grafting and antibiotics. *Singapore Medical Journal*.

[4] Ip K. C., Lam Y. L., Chang R. Y. P. (2008). Brodie's abscess of the ulna caused by *Salmonella typhi*. *Hong Kong Medical Journal*.

[5] Tan K., Yoong P., Marshall T. J., Martin C. (2012). Percutaneous drainage as a novel approach for the treatment of Brodie's abscess. *Clinical Radiology*.

[6] Al-Jafar H., Al-Shemmeri E., Al-Shemmeri J., Aytglu L., Afzal U., Al-Enizi S. (2015). Precision of SPECT/CT allows the diagnosis of a hidden brodie’s abscess of the talus in a patient with sickle cell disease. *Nuclear Medicine and Molecular Imaging*.

[7] Buck A. K., Nekolla S., Ziegler S. (2008). SPECT/CT. *Journal of Nuclear Medicine*.

